# Trifecta in flexible ureteroscopy for treatment of renal and upper ureteral calculi: A multicenter study

**DOI:** 10.1080/20905998.2024.2325784

**Published:** 2024-03-10

**Authors:** Ahmed R. EL-Nahas, Moustafa G. Elhammadi, Ahmed E. Abolazm, Mahmoud N. Laymon, Ehab R. Tawfiek, Mohamed El-Shazly, Ahmed M. Elshal, Ramy Elbaz, Ahmed B. Shehab El-Din, Ahmed M. Shoma

**Affiliations:** a Department of Urology, Urology and Nephrology Center, Mansoura University, Mansoura, Egypt; b Department of Urology, Minia University, Minia, Egypt; c Department of Urology, Menoufia University, Menoufia, Egypt

**Keywords:** Flexible ureteroscopy, trifecta, RIRS, renal calculi, stones

## Abstract

**Purpose:**

To determine predictors for missing trifecta in patients who underwent flexible ureteroscopy (FURS) for treatment of renal and upper ureteric calculi.

**Patients and Methods:**

The data of adult patients with renal or upper ureteral stones who underwent FURS from June 2021 through December 2022 were retrospectively reviewed. Stone-free status (no residual stones > 3 mm) was evaluated after 3 months with non-contrast CT. Modified Clavien classification was used to grade complications. A stone-free status after a single intervention of FURS without complications was defined as trifecta. Patients were divided into two groups (trifecta and non-trifecta). Risk factors for missing trifecta were compared between both groups using univariate and multivariate analyses.

**Results:**

Three hundred twenty-three patients with mean age 48.9 ± 13 years and mean stone length 16 ± 5.9 mm were included. The trifecta criteria were applicable for 250 patients (71%). On multivariate analysis, risk factors for missing trifecta were stone multiplicity (OR: 3.326, 95%CI: 1.933–5.725) and non-experienced surgeons (OR: 1.819, 95%CI: 1.027–3.220).

**Conclusions:**

Multiple stones and performance of FURS by non-experienced surgeons are the independent risk factors for missing trifecta of FURS.

## Introduction

Urolithiasis is a common disease that implies a major clinical and economic burden for healthcare systems. It can compromise renal function and leads to chronic kidney disease (CKD) and renal failure [1].

Flexible ureteroscopy (FURS) is a safe and effective treatment for renal stones when compared to percutaneous nephrolithotomy (PCNL). However, re-treatment and the requirement for auxiliary procedures were more after FURS [2]. FURS was proved superior to shockwaves lithotripsy (SWL) for lower pole calculi <20 mm [3]. The European Association of Urology (EAU) guidelines on urolithiasis recommend FURS for the treatment of renal stones of <20 mm that are not suitable for SWL for various reasons such as unfavorable pelvicalyceal anatomy or stone characteristics, bleeding disorders, or due to occupational or social factors [4].

The use of FURS for treatment of renal stones has been widely extended with advancements in endoscopic technology and laser systems. However, the first recommended treatment modality for renal stones >20 mm is PCNL. The current surgical techniques in FURS and laser lithotripsy make it a feasible treatment option in the hands of experienced surgeons [5]. However, multiple sessions are usually needed to achieve the stone-free status, avoid a long operative time, and decrease the risk of sepsis. One of the methods used to decrease operative time was using silodosin for 10 days before FURS [6].

Evaluation of the outcomes of minimally invasive treatment modalities for management of renal calculi relied on many parameters such as stone-free status, complications, and number of treatment sessions. Recently, the new ‘Trifecta’ tool was set in motion to help standardize reporting of stone intervention outcomes. Trifecta for stone treatment was defined as stone-free status in a single session without complications [7]. Then it was externally validated for mini-PCNL [8].

This study was conducted to determine predictors for missing trifecta in patients who underwent FURS for treatment of renal and upper ureteral calculi.

## Patients and methods

The study protocol was approved by the local medical research ethics committee (Code: MS.23 February 2291). The electronic data of patients who underwent FURS from June 2021 through December 2022 in three hospitals were retrospectively reviewed. All consecutive adult patients with renal or upper ureteric stones that were indicated for FURS according to AUA guidelines were included. Patients with incomplete follow-up were excluded. Preoperative workup included laboratory tests (urinalysis and culture, serum creatinine, CBC, and INR). Non-contrast CT (NCCT) was used to evaluate stone characteristics and distribution in the kidney.

### Technique of flexible URS

After spinal anesthesia, patients were placed in lithotomy position. For patients with preoperative sterile urine cultures, an IV dose of third-generation cephalosporin antibiotic was used within one hour of FURS. Patients with infected preoperative urine cultures received 5 days of specific antibiotics according to sensitivity test. The procedure started with a retrograde study through a ureteric catheter. Then, a guidewire was introduced through the catheter to coil in the renal pelvis. A dual lumen catheter was introduced over this guidewire, and a second one was passed till reaching the calyceal system. Flexible ureteroscopy was performed using a 9.5 F reusable flexible ureteroscope (FlexXc™, Karl Storz Endoscope, Tuttlingen, Germany) or single-use flexible URS (LithoVue™, Boston scientific, Massachusetts, USA). According to surgeon preference, the FURS passed over the second guidewire or through an 11/13 F ureteral access sheath (UAS). Stones were disintegrated using Holmium: YAG laser machine (Sphinx Jr, LISA Laser Products, Katlenburg-Lindau, Germany). Laser settings were 0.5 Joule and 20 Hz for dusting or 1 Joule and 10 Hz for disintegration. Stone fragments were retrieved by 1.9 F. Zero tip basket (Boston Scientific, Spencer, IN, USA) when needed. A 6 F. ureteric stent or ureteric catheter was placed at the end of the procedure as indicated.

### Outcomes evaluation

Stone-free status was evaluated after 3 months with NCCT of the abdomen and pelvis. Stone-free was defined as no residual stones >4 mm. Complications were graded according to modified Clavien classification. Trifecta was defined as stone-free outcome without complications after a single session of FURS. Patients were divided into two groups (trifecta and missing trifecta). The missing trifecta group included patients who had significant residual stones, complications, or underwent more than one session of FURS or auxiliary procedure. Factors that may affect the outcome were compared between both groups. Preoperative factors included age, gender, creatinine, urine culture results, stone length, side, and distribution in pelvi-calyceal system. Stone length was the maximum diameter of the stone as measured in axial and coronal images of NCCT. Tested operative factors were the use of ureteral access sheath, laser mode used (dusting or fragmentation), and retrieval of stone fragments after disintegration. Experienced surgeons were defined as those who have done more than 100 FURS cases [9].

### Statistical analysis

Patients’ data were stored and analyzed by SPSS v20 software (IBM SPSS Statistics, Armonk, NY). Factors affecting trifecta were compared between both groups by univariate (chi-square or t-test) and multivariate (binary logistic regression) analyses to detect risk factors. *p* value < 0.05 was set for statistical significance.

## Results

The study included 323 patients. **Baseline characteristics of all patients are presented in** Table 1. Mean operative time was 72.4 ± 23.6 minutes, and mean postoperative stent duration was 20 ± 9.7 days. It was observed that experienced surgeons performed FURS for 97% of stones larger than 20 mm.

**Table 1.** Baseline characteristics and overall outcomes of all patients.

	323 Patients
**Categorical Variables**	**N. (%)**
Sex: Male Female	185 (57.3) 138 (42.7)
UTI: No Yes	242 (75) 81 (25)
Stone Recurrence: No Yes	251 (77.7) 72 (22.3)
Stone Side: Right Left	164 (50.8) 159 (49.2)
Stone Multiplicity: Single Multiple	209 (64.7) 114 (35.3)
Stone Length: <20 mm 20 mm and more	235 (72.8) 88 (27.2)
Hydronephrosis: No Yes	283 (87.6) 40 (12.4)
Preoperative Stent: Yes No	164 (50.8) 159 (49.2)
Solitary Kidney: No Yes	281 (87) 42 (13)
Complications: No Yes	295 (91.3) 28 (8.7)
Stone-Free: Yes No	278 (86) 45 (14)
Continuous Variables	Mean (SD)
Age (years)	49 (13)
BMI (kg/m^2^)	30.2 (5.8)
Creatinine (mg/dl)	1.3 (0.6)
Stone Length (mm)	16 (5.9)
Stone Density (HU)	922 (371)
Operative Time (min.)	72.4 (23.6)
Hospital days	1.9 (1.1)

Intraoperative ureteral wall injuries were inflected in four patients and were successfully managed with ureteric stenting for 4 weeks. Postoperative complications included septicemia in two patients and acute coronary syndrome in one patient requiring intensive care unit admission (Grade 4). Postoperative fever (>38.5°Celsius) was recorded in 19 patients and was managed with antibiotics and antipyretics (Grade 2). Transient hematuria for three days was encountered in five patients who were managed conservatively without blood transfusion (Grade 1). Three patients had both intraoperative and postoperative complications; therefore, the total number of patients with complications was 28 (8.7%, Table 1).

It was found that 8% (26 patients) required two procedures in the form of FURS in 17, SWL in 5 and PCNL in 2 patients. As shown in Table 1, stone-free status at 3 months was 86% (278 patients). The trifecta criteria were applicable for 250 patients (71%). Univariate analyses for risk factors of missing trifecta are presented in Table 2. On multivariate analysis, independent risk factors were stone multiplicity and non-experienced surgeons (Table 3).

**Table 2.** Univariate analysis for risk factors of non-trifecta after flexible ureteroscopy.

	Trifecta 250 Patients	No Trifecta 73 Patients	
Categorical Variables	N. (%)	N (%)	P *
Sex: Male Female	140 (75.7) 110 (79.7)	45 (24.3) 28 (20.3)	0.391
UTI: No Yes	192 (79.3) 68 (71.6)	50 (20.7) 33 (28.4)	0.150
Stone Recurrence: No Yes	200 (79.7) 50 (69.4)	51 (20.3) 22 (30.6)	0.067
Stone Side: Right Left	126 (76.8) 124 (78)	38 (23.2) 35 (22)	0.804
Stone Multiplicity: Single Multiple	178 (85.2) 72 (63.2)	31 (14.8) 42 (36.8)	<0.001
Stone Site: Renal Pelvis Lower Calyx Middle Calyx Upper Calyx Pelvis and Calyceal Multiple Calyceal Upper Ureter Ureter and Calyceal	67 (81.7) 57 (81.4) 20 (80) 16 (84.2) 22 (59.5) 19 (67.9) 29 (87.9) 20 (69)	15 (18.3) 13 (18.6) 5 (20) 3 (15.8) 15 (40.5) 9 (32.1) 4 (12.1) 9 (31)	0.058^#^
Stones in Multiple Sites: No Yes	189 (82.5) 61 (64.9)	40 (17.5) 33 (35.1)	0.001
Stone Length: <20 mm 20 mm and more	178 (75.7) 72 (81.2)	57 (24.3) 16 (18.2)	0.245
Hydronephrosis: No Yes	218 (77) 32 (80)	66 (23) 8 (20)	0.674
Preoperative Stent: Yes No	128 (80.5) 122 (74.4)	31 (19.5) 42 (25.6)	0.189
Solitary Kidney: No Yes	218 (77.6) 32 (67.2)	63 (22.4) 10 (23.8)	0.841
UAS: No Yes	192 (76.5) 58 (80.6)	59 (23.5) 14 (19.4)	0.468
Laser Lithotripsy: Dusting Disintegration	216 (77.4) 34 (77.3)	63 (22.6) 10 (22.7)	0.983
Fragments Retrieval: No Yes	196 (77.5) 54 (77.1)	57 (22.5) 16 (22.9)	0.954
Surgeon Experience: Experienced Non-Experienced	187 (80.6) 63 (69.2)	45 (19.4) 28 (30.8)	0.028
Continuous Variables	Mean (SD)	Mean (SD)	P^&^
Age (years)	48.7 (12.8)	49.7 (13.9)	0.558
BMI (kg/m^2^)	30.2 (5.8)	30.1 (5.5)	0.877
Creatinine (mg/dl)	1.3 (0.6)	1.1 (0.4)	0.060
Stone Length (mm)	15.8 (6.1)	16.5 (4.7)	0.340
Stone Density (HU)	907 (375)	977 (356)	0.158

*: Chi-square test, ^#^: Fishers exact test, ^&^: Independent sample t-test.

**Table 3.** Multivariate (logistic regression) analysis for factors predicting non-trifecta for flexible URS.

Independent Risk Factor	Odds Ratio	P Value	95% Confidence Interval (Lower – Upper)
Stone Multiplicity: Single (Reference) Multiple	3.326	<0.001	(1.933–5.725)
Surgeon Experience: Experienced (Reference) Non-experienced	1.819	0.040	(1.027–3.220)

## Discussion

Trifecta in evaluation of stone treatment was introduced by EL-Nahas et al. for mini-PCNL [7] and externally validate by Pozzi et al. [8]. The advantage of this tool of outcome evaluation is the ability to combine safety and efficacy for a specific stone treatment procedure. Moreover, it depended on both surgeon- and patients’-centered outcomes as the surgeon is interested in stone-free status without complications while the patient is interested in avoiding complications and being free of stones after one intervention. Therefore, it can be applied for any procedure used for upper tract stone treatment (such as PCNL, FURS, or SWL). It was used in the present manuscript for FURS. Trifecta was achieved in 71% of cases which is lower than previously reported trifecta of mini-PCNL (84%) [7]. In a meta-analysis comparing Mini-PCNL and FURS, stone-free rates were superior after mini-PCNL [2].

The risk factors for missing trifecta include multiplicity of the stones and performance of the procedure by non-experienced surgeon. It is obvious that multiple stones are the reason for the need of multiple sessions or significant residual stones while non-experienced surgeon may the cause for more complications [9]. Surgeons working in high-volume centers get FURS experience rapidly, and this was reflected on more stone-free rates, lower need for retreatment, and lower rate of complications when compared with those working in low-volume centers [10]. The lower complications after FURS that were performed by experienced surgeons were proved by Berardinelli et al. in a propensity score analysis. Complications were significantly lower with experienced surgeon in comparison with non-experienced surgeons (*p* = 0.001) [9]. Moreover, experienced surgeons tend to perform FURS for larger stones [11]. Frontczak et al. performed propensity score-matched retrospective study and found that stone-free status for large renal stones (>20 mm) was significantly better with experienced surgeons [12]. The same was observed in the present study as 97% of large stones were treated by experienced surgeons, and therefore, trifecta was better for these large stones.

In this study, the stone-free status was 86%. Recent meta-analyses revealed a stone-free rate ranging between and 80% and 89% when using FURS in treating upper urinary tract stones [13,14]. One of them confirmed a lower stone-free status in case of multiple stone locations [14]. In the present study, stones in multiple locations were significant only in univariate analysis. There are multiple trials to improve outcomes of FURS. Recently, flexible-tip ureteral access sheath with suction was proved to increase the stone-free rate and decrease complications of FURS in treatment of renal calculi [15]. Diab et al. showed the preoperative silodosin resulted in decreased incidence of ureteric injury during FURS [16]. Another study showed that the RIRS stone complexity score could predict stone-free status after FURS, but no scoring system could predict complications [17].

In the present study, 8% of patients needed re-treatment with FURS, SWL, or PCNL. Zewu et al., in their meta-analysis, found that 39% of patients treated with FURS needed complementary procedures to achieve a stone-free rate comparable to PCNL [18]. This discrepancy may be related to the fact that the mean stone length in our study is 16 mm while in this meta-analysis they included larger stone sizes between 2 and 3 cm.

Davis et al. reported complication in 15.5% (range 5–84%) in their meta-analysis of 16 FURS studies [14]. In this study, complications were encountered in 8.7%. This was in the range of previous reports. It was also observed, in this study and other reports, that most of these complications were low grades (grades 1 and 2). In our study, overall infectious complications were evident in 21 patients (6.5%). Similar rates were reported in a systematic review of 17 studies that showed an average infectious complications rates of 2.8–7.5% [19].

This study is limited by its retrospective design that may imply selection bias. Another limitation is lack of postoperative stone analysis. However, the strength of it is the multicenter evaluation of the results that utilized the data of three different hospitals from different locations.

## Conclusions

Multiple stones and performance of FURS by non-experienced surgeons are the independent risk factors for missing trifecta of FURS. **Future prospective studies may help with better evaluation of more possible factors that affect achieving trifecta with FURS.**
